# The Role of Attachment Security in Adult Children’s Caregiving for Parents With Cognitive Impairment Over Time

**DOI:** 10.1093/geroni/igaf122.3671

**Published:** 2025-12-31

**Authors:** Angeliki Karachaliou, Mary O’Dea, Joan Monin

**Affiliations:** Yale School of Public Health, new haven, Connecticut, United States; Yale School of Public Health, New Haven, Connecticut, United States; Yale, Yale, Connecticut, United States

## Abstract

Having a parent who is beginning to experience cognitive impairment is a challenging time for adult children both physically and emotionally. Yet, little is known about the potential positive aspects of caregiving including viewing the role of caregiving as rewarding and enriching individuals’ life experiences at this stage of a parent and adult child’s relationship. One theoretical framework which could explain the variance in caregiver perceptions is attachment theory. Attachment styles of emotional bonding, based on the nature of early emotional connections with parental figures, persist into adulthood impacting the way an individual later receives and provides care. Two main patterns characterize insecure attachment: anxiety and avoidance. People who score low on both are considered securely attached, which is thought to be the most adaptive to stress. Our study consisted of 150 early-stage cognitively impaired parents and one adult child who completed surveys at baseline and one year later, and the aim was to examine whether the adult child’s attachment anxiety and avoidance to their parent with cognitive impairment was associated with lower positive views of caregiving. Results from the statistical model showed that only avoidant attachment was associated with lower positive experiences on caregiving at baseline. Attachment dimensions at baseline did not predict year two caregiving positivity controlling for baseline attachment dimensions. As attachment and familial care in later life remains a highly under-researched area, understanding the factors forming the caregivers’ experiences early in the caregiving journey is critical to design effective psychosocial interventions and better support parent-child dyads.

